# The performance evaluation of a urine malaria test (UMT) kit for the diagnosis of malaria in individuals with fever in south-east Nigeria: cross-sectional analytical study

**DOI:** 10.1186/1475-2875-13-403

**Published:** 2014-10-15

**Authors:** Tagbo Oguonu, Elvis Shu, Bertilla U Ezeonwu, Bao Lige, Anne Derrick, Rich E Umeh, Eddy Agbo

**Affiliations:** Department of Pediatrics, University of Nigeria Teaching Hospital, Enugu, Nigeria; Consortium for Health Research and Advanced Training, College of Medicine University of Nigeria Enugu Campus, Enugu, Nigeria; Department of Pediatrics, Federal Medical Center, Asaba, Nigeria; Fyodor Biotechnologies Inc, 800-West Baltimore Street, Baltimore, MD 21201 USA

**Keywords:** Diagnostic kit, Malaria, Nigeria, Sensitivity, Specificity

## Abstract

**Background:**

Accurate rapid diagnosis is one of the important steps in the effort to reduce morbidity and mortality of malaria. Blood-specific malaria rapid diagnostic tests (RDTs) are currently in use but other body fluid specific diagnostic test kits are being developed. The aim of the present study was to evaluate the performance characteristics of a one-step Urine Malaria Test™ (UMT) dipstick in detecting *Plasmodium falciparum* HRP2, a poly-histidine antigen in urine of febrile patients for malaria diagnosis.

**Methods:**

This was an observational study in which a urine-based malaria test kit was used in malaria diagnosis in a normal field setting. Two hundred and three individuals who presented with fever (≥37.5°C) at seven outpatient clinics in Enugu State during periods of high and low transmission seasons in Southeastern Nigeria were enrolled. Matched samples of urine and blood of consecutively enrolled subjects were tested with UMT and blood smear microscopy.

**Results:**

With the blood smear microscopy as standard, the disease prevalence was 41.2% and sensitivity for the UMT was 83.75% (CI: 73.81 to 91.95%, Kappa 0.665, p =0.001). The UMT had an LLD of 120 parasites/μl but the sensitivity at parasite density less than ≤200 parasites/μl was 50% and 89.71% at density ≥201 parasites/μl with specificity of 83.48%. The positive and negative predictive values were 77.91% and 88.07%, respectively.

**Conclusion:**

The UMT showed moderate level of sensitivity compared with blood smear microscopy. The test kit requires further improvement on its sensitivity in order to be deployable for field use in malaria endemic regions.

## Background

Malaria causes significant morbidity and mortality especially among children in sub-Saharan Africa. The disease is caused by the *Plasmodium* species*,* which is transmitted to humans by the bite of the female *Anopheles* mosquito*.* It is estimated that malaria causes about 207 million clinical episodes and about 627,000 deaths annually, mostly in sub-Saharan Africa [1]. One of the factors that have ensured the persistence of malaria and the morbidity/mortality has been the failure of early diagnosis and prompt treatment of the disease [2, 3]. Until recently, presumptive diagnosis based on clinical algorithm in highly endemic areas has been the most used method [4, 5], but is been found unreliable and contributed to over-diagnosis of malaria, leading to wastage of anti-malarial drugs [6].

However, in recent times, with relatively high cost and increasing incidence of resistance to anti-malarial drugs, it has become necessary to confirm cases of malaria using laboratory methods before treatment. Thick smear microscopy based on the demonstration of the presence of *Plasmodium* parasite in blood remains the gold standard for the diagnosis of malaria [4].

However, this method is highly operator-dependent and requires initial and ongoing training to maintain high quality testing; such quality assurance practices have often been difficult to implement in resource poor and malaria endemic countries. In addition, there has been reported over-diagnosis of malaria using microscopy in health facilities [6, 7], with its consequent effect on drug resistance and toxicity. To reduce these deficiencies, the malaria rapid diagnostic test (malaria RDT) kits have been developed and deployed in many countries for the rapid diagnosis of clinical malaria.

These kits use immune-chromatographic materials impregnated with monoclonal antibodies against *Plasmodium* species to detect the parasite antigen in blood of infected patients. Most of these kits use antigens or enzymes derived from parasites that have infected humans. The commonly used one is the histidine rich protein 2 (HRP2), which is produced by *Plasmodium* during its asexual forms and early gametocytic stages in infected individuals. It is a water soluble substance abundantly found in the parasite cytoplasm and serum of infected individuals. Histidine rich protein 2 is secreted early during infection by the *Plasmodium* parasite and persists after treatment. Significant levels of HRP2 antibodies in some infected individuals reduce the sensitivity of the protein dependent test kits [4]. Sensitivities of the HRP2 kits are affected by factors which are dependent on the protein, the individual and manufacturing. Environmental factors such as high temperatures are equally known to affect sensitivities [4]. Over time, the HRP2 based rapid diagnostic test kits have improved on their sensitivity and specificity to the ranges of 80% to 95% compared with malaria microscopy [8]. In order to standardize the efficacy of the malaria RDT kits, the WHO set the lowest level of detection (LLD) acceptable in areas of high malaria endemicity for all malaria RDTs to be 100 parasites/μl [9], However, due to poor correlation noted between the low parasite density (<100 parasites/μl) and RDTs, another point of 200 parasites/μl was set as standard for diagnostic test kits for field tests [10].

HRP2 have been found in several body fluids such as urine, saliva, blood [11]. These findings increased interest in the use of malaria RDTs for diagnosis of malaria in these types of body fluids. With concerns about blood-borne pathogen exposures, cultural taboos and procedural difficulties, the advantages of the use of urine as test matrix include low risk and relative ease of access of the sample. Blood-based malaria RDTs have been evaluated for malaria detection in urine with unsatisfactory results [12, 13]. The performance may be attributed to the degradation or proteolytic cleavage of urine-excreted proteins [14, 15], which may necessitate the use of appropriately specific kits that can identify specific malaria antigens in the urine. Recently, a urine based malaria test kit (UMT) has been developed by Fyodor Biotechnologies Baltimore, USA for the diagnosis of malaria. The UMT is a recombinant monoclonal antibody based test that detects *Plasmodium falciparum* specific HRP2, a poly-histidine protein, or fragment present in the urine of febrile patients.

The present study was performed to assess the diagnostic accuracy of the UMTin malaria diagnosis in comparison with benchmarked gold standard, blood smear microscopy.

## Methods

This was an observational cross-sectional diagnostic test study in which participants with fever at presentation were enrolled consecutively at different health facilities in both urban and rural communities of Enugu State. Institutional ethical approval was obtained from the University of Nigeria Teaching Hospital Health Research Ethics Committee prior to the start of the study. Individuals who met inclusion criteria were properly consented before enrollment.

### Study site

Enugu State has a population of 3.6 million inhabitants and located in the rain forest region of southeast Nigeria. The region is endemic for malaria and with year-round transmission of the disease. The highest transmission is in the rainy season from the month of April to October.

The inclusion criteria were: fever of ≥37.5°C and non-use of anti-malarial drugs in the preceding one week. Adults and children with history of haematuria were excluded from the study (haematuria is a feature of many diseases with probable high level of antibody that may cause false positive results.). Demographic information such as age, gender, symptoms of disease as well as physical examinations were also obtained from each participant. Subsequently the matched capillary blood drawn from finger/heel pricks and urine samples voided into a universal bottle were respectively stained in accordance with WHO standard microscopy technique [16] and immediately tested with the UMT kit (Lot No. K06K-0, Fyodor Biotechnologies, Baltimore, Maryland, USA).

### Principles and procedure for Urine Malaria Test™ (UMT)

The technology relies on the fact that clinical malaria commonly results in elevated levels of specific proteins (Histidine Rich Protein-2) or its protein fragments in patient’s urine against which cognate recombinant monoclonal antibody reagents were developed. It is a qualitative assay consisting of a nitrocellulose membrane strip containing relevant antibody reagent and controls that are each immobilized at the specific individual site on the membrane. When the immunochromatographic dipstick is dipped in urine, specific malaria parasite protein present in the urine migrate and interacts with immobilized cognate monoclonal antibody resulting in dark-colored strips on the dipstick. The UMT strips were individually packaged in a sealed Mylar foil pouch with a desiccant, and stored at room temperature for the entire period of the study.

### Sample testing

To perform the urine test, the UMT strip was dipped in 200 μl of urine for two minutes to allow the sample to wick and saturate the strip. The strip was then removed, placed on its foil pouch packaging and incubated for 20 minutes. The results were reported as negative, positive, or un-interpretable: if two visible lines appeared on the strip (even if very faint) the test was positive; if only the control line appeared, the test was negative. Tests results reported as un-interpretable, i.e., failure to observe a control line or the presence of a darkly stained background that obscured the test lines, were repeated to resolve the discrepant event.

### Blood microscopy

The capillary blood samples drawn from finger/heel pricks of the subjects were used to perform thick smear microscopy. Preparation of these blood samples (three thick blood smear slides per participant) for microscopy were in accordance with WHO standard microscopy technique [16], and read with × 1,000 magnification (with oil immersion lens). Two trained and experienced microscopists at the laboratory read the slides independently. Microscopy was considered positive only when asexual parasite forms – trophozoites and schizonts (not gametocytes alone) – were detected, since asexual forms are indicative of active infection. Parasite densities were determined by counting the number of parasites seen per 200 white blood cells, and the parasite density per microlitre was calculated based on the putative mean count of 8,000 leucocytes per microlitre using the formula [4, 16]:


However, if 500+ parasites have been counted without having reached 200 leucocytes, the count was stopped after completing the reading of the last field, and the parasitemia was calculated using the above formula. Also 100 fields of the second thick film were examined to identify mixed infections, which were confirmed on the thin film in case of any doubt. A blood film was assumed negative when the examination of 100 thick film fields did not show the presence of asexual forms of *P. falciparum.* The same technique was employed for establishing the parasite density on each of the subsequent blood film examinations.

To yield a final microscopic interpretation, the two study microscopists independently agreed on three criteria: (i) on the presence or absence of asexual stages of *Plasmodium*; (ii) on the species of *Plasmodium*, when one was present; and (iii) on the calculated level of parasitaemia within a 10% error margin [17, 18].

### Data management and analysis

All clinical and laboratory data were entered into the corresponding logbooks and transcribed into Excel worksheet (Windows 7, Microsoft Inc., Richmond, Washington 2011) and analysed with Statistical Package for Social Science (SPSS) version 20. (IBM Inc. Chicago, Illinois). Descriptive statistics were used to ascertain the frequency distribution, mean, median and standard deviation of the subject characteristics; age, temperature, clinical symptoms. The outcome variables: the parasite densities and the UMT results were expressed in proportions (percentages), categorized for determination of sensitivity, specificity and predictive values (positive and negative). Chi square tests were used to test for the significance of the UMT results and other subjects’ derived categorical variables. P value of 0.05 was set for statistical significance.

## Results

### Patient enrollment

From a total of 638 individuals who were screened, 203 who met the inclusion criteria were enrolled from all the health facilities between June and December 2012, spanning periods of high and low malaria transmission in the study area. Data from 203 subjects were analysed; three children who could not void urine and five that had incomplete information were excluded giving final number of 195 subjects. Sixty four percent of the participants were females and 90.3% were children (aged less than 18 years old). The median age was three years with the range of six months to 60 years (Table 1). All the participants presented with fever (≥37.5°C) at enrollment, with a median body temperature of 38.1°C, (mid quartile range: 37.7°C to 38.9°C).

**Table 1:** **The demographic characteristics and parasite densities of the study population**

General characteristics	
**Age (SD) years**	6.80 (11.45)
**Median (quartile range) years**	3 (3 to 5.91)
**Age groups (%)**	
< 5 years	144 (73.8%)
5-18 years	31 (15.9%)
>18 years	20 (10.3%)
**Species Identification (%)**	
*Plasmodium falciparum (Pf)*	66 (30.4%)
*Pf and P vivax*	125 (64.1%)
**Geometric mean parasite density**	62, 778.85
parasites/μl	
Grouped Median (range)	9,080 (60 to 792, 600
parasites/μl	

SD = Standard deviation.

### Malaria microscopy

Eighty (41.03%) out of the 195 participants had positive malaria *P. falciparum* parasites with microscopy Table 2. The parasite infection rates with blood smear microscopy among children and adults screened were 37.43% and 3.58%, respectively. The parasite density geometric mean was 62,778.9 parasites/μl with a range of 60 to 792,600 parasites/μl and grouped median of 9080 parasites/μl. The microscopy specie identification showed *P. falciparum* in blood specimen of all the subjects out of which 64.1% had a mixed infections of *P. falciparum* and *Plasmodium vivax.*

**Table 2:** **UMT and Blood microscopy test results for malaria parasites**

		MICROSCOPY		Total
		Positive	Negative	
UMT	Positive	67	19	86
	Negative	13	96	109
Total		80	115	195

### Urine malaria test

Eight-six (44.1%) of 195 were positive with the UMT test. Parasite rates were 41.03% and 3.0% among children (<18 years) and adults, respectively. Overall, the UMT in comparison with the malaria microscopy showed sensitivity of 83.75% (95% CI: 73.81 to 91.95%, Kappa 0.665, p =0.001) and specificity of 83.48% (95% CI: 75.41% to 89.75%), Table 3. The device showed different sensitivities for different malaria parasite densities (PD) as follows: PD ≤100 parasites/μl: 0%, PD ≤200 parasites/μl: 50%, PD ≥201 parasites/μl: 89.71%, respectively Table 3. The lowest parasite density detected was 120 parasites/μl. Two of the urine samples showed a negative UMT result in the presence of high malaria parasite densities (61,520 and 100,269 parasites/μl) with microscopy. There were 19 false positive results of which one was due to the presence of gametocyte alone in the blood smear. Indeterminate results were observed in one test which on repeat showed a faint positive result. Most commonly observed was the degradation of the test results over time (within one hour post-test).

**Table 3:** **Sensitivity of UMT with microscopy at different parasite densities**

	Parasite densities (parasites/μl)			
Parameters	≤ 100	≤ 200	≥ 201	Overall
Sensitivity	0	50%	89.71%	83.75%
95% CI	0% to 80.71%	21.21% to 78.79%	79.93% to 95.74%	73.81% to 91.05%
PPV	0%	24%	76.25%	77.91%
95% CI	0.00% to 17.80%	9.42% to 45.13%	65.42% to 85.05%	67.67% to 86.14%
NPV	97.96%	94.12%	93.20%	88.07%
95% CI	92.81 to 99.69%	87.63% to 97.80%	86.49% to 97.41%	80.47% to 93.49

Among the different age groups, false positive results were less frequent among the adults than the children (8.3% versus 17.14%, p = 0.001), while the false negative results were less among the children than adults (12.12% versus 28.57%, p = 0.001). Overall, the positive predictive (PPV) and the negative predictive values (NPV) for the UMT were 77.91% and 88.07%, respectively.

## Discussion

The UMT prototype has shown a higher level of sensitivity than previously tested blood specific RDT kits in urine specimens [12, 13]. Genton *et al.* using ParaSight^®^-F to test urine samples of malaria patients obtained sensitivity and specificity of 81% and 26%, respectively [13]. Other studies as well with non-blood samples showed comparatively poorer sensitivity results: saliva (73%) and urine (32%) [13]. The UMT had an LLD of 120 parasites/μl, and a 50% sensitivity at ≤200 parasites/μl which is the revised acceptable standard for field tests [10, 19] it however requires further improvement despite the corresponding good specificity. There could be many reasons for the relative poor sensitivity at lower parasitaemia levels, which may be related to parasite antigen production, antigen content in urine and time of void [20, 21]. Genton *et al.* [12] noted that the amount of malaria antigen was low in urine and dependent probably on the time of collection of the samples [12]. They suggested that first void morning urine might probably give better sensitivity than later timed samples. This may not be practicable in clinical practice where the results are required for immediate treatment. With the probable variability in malaria antigen quantity, it is likely that the expected amount of antibody impregnated in the urine-specific test kits as well as the quantity of body fluid required may be higher than those of blood-specific test kits thus necessitating a probable further optimization of the Fyodor UMT to enhance test sensitivity in low parasitaemia. It is known that the property of the antibody impregnated in the nitrocellulose pad of the immunochromatographic test kits also determine the sensitivity. For instance the monoclonal or polyclonal antibodies affect the degree different of the malaria RDTs [20]. At higher parasite densities (≥200 parasites/μl of blood) the UMT sensitivity and specificity in urine were comparable with the results of blood based test kits [4, 21] in blood samples.

The degree of false positive results especially among children may suggest that the UMT is able to maintain acceptable level of false positive results particularly in areas of high malaria transmission [22, 23]. Also the false positivity related to the presence of the gametocyte is indicative of the ability to detect sexual form of *P. falciparum* a factor which is useful in absolute sensitivity tests against the clinical episodes that was used in this study. However, in areas of low malaria endemicity, this level of false positives may create drug wastage, which the current malaria control efforts seek to reduce. False positive results may be attributed to the ability of all histidine-rich protein 2 (HRP2) antigen malaria test kits to detect the parasite antigen even after malaria illness. The presence of rheumatoid factor and schistosomiasis in a patient may also lead to false positivity, and will need to be further evaluated [24–26]. These factors are known to affect the blood type malaria RDTs, but little is known about such influence on the urine malaria test kits. It may be assumed that since both (blood and urine-based) test kits are specific for HRP2 such effect may also occur with the UMT.

The UMT specificity remained constant at significant levels for the different parasite densities although this may cause significant morbidity and mortality in areas of high endemicity. The false negative results with UMT are comparable to those of blood-specific malaria RDTs [4, 11, 12, 21]. Many factors have been described to contribute to the false negative results with HRP2-based rapid test kits. These include parasite and host factors such as deletion or mutation of HPR2 gene [27], the presence of antibodies to HRP2 in the presence of high malaria density [4, 27]. The two cases noted in this index study that had high malaria parasite count greater but with negative UMT results may be an illustration of the prozone effect observed with immunochromatographic tests such as malaria RDT [28–30].

Some of the limitations observed with the use of the urine malaria test kit were the high color degradation of the test line within an hour after testing. This is a disadvantage, which makes it difficult to crosscheck the results in cases of doubt. The delay in provision of some subjects, particularly among children, may be a delay factor in the promptness of testing and treatment. The sample size may also may be a factor in the resultant accuracy result, which may probably be answered by the completed larger clinical trials.

## Conclusion

The Urine Malaria Test kit studied has shown moderate concordance with blood smear microscopy and higher levels of sensitivity than blood-based test kits used for malaria diagnosis with urine. The UMT used for the study is still undergoing further development and evaluation, with opportunity for further improvement particularly in the area of test stability and sensitivity at lower parasite levels before the kit can be fully deployed for general use. Optimization of the detected areas of deficiency will enhance the usefulness of the non-blood based kits such as the UMT as a reliable and easy to use alternative tool in the diagnosis of malaria in the areas where the competence for blood microscopy is not reliable, and safety concerns are expressed.
